# First description study on molecular characterization of *Hemiclepsis khankiana* (Hirudinea: Glossiphoniidae) in China

**DOI:** 10.1186/s12917-023-03796-w

**Published:** 2023-11-18

**Authors:** Yongcai He, Zengkui Li, Wenjie Jin, Changzhong Li, Guojie Wang, Zhengji Wang, Ming Kang, Jiquan Li, Jianlin Wang, Xiaoyu Hu, Shuo Jiang, Qiang Chen, Haolin Li, Dong Gao, Peiqi Liu, Ying Li

**Affiliations:** 1 College of Agriculture and Animal Husbandry, Qinghai University, Xining, 810016 Qinghai China; 2 College of Eco-Environmental Engineering, Qinghai University, Xining, 810016 Qinghai China; 3 Qinghai Provincial Fishery Environmental Monitoring Center, Xining, 810012 Qinghai China; 4 Qinghai Institute for Endemic Disease Prevention and Control, Xining, 811602 Qinghai China; 5 Key Laboratory of Plateau Cold-water Fish Culture and Eco-environmental Conservation (Co-construction by Ministry and Province), Ministry of Agriculture and Rural Affairs, Qinghai University, Xining, 810016 China; 6 Qinghai Provincial Key Laboratory of Pathogen Diagnosis for Animal Diseases and Green Technical Research for Prevention and Control, Xining, 810016 China

**Keywords:** Leech, Glossiphoniidae, *Hemiclepsis khankiana*, China

## Abstract

**Background:**

Leeches are an integral component of aquatic biocenosis and can be found in a wide range of ecosystems such as freshwater, saltwater, flowing, and still-water ecosystems. It especially plays an important role in the freshwater benthic community and is an important part of the food web. In this study, a leech species was found in the mantle cavity of wild freshwater mussels in Zigong City, Sichuan Province, China, and its identity was determined through morphological analysis and molecular biological analysis.

**Results:**

The leech is *Hemiclepsis khankiana*, a new species of *Hemiclepsis* that has been discovered in Russia in recent years. Through morphological analysis, the current survey observed that the morphological characteristics of *Hemiclepsis khankiana* eyespots were significantly different from the first reported description. The first pair of eyespots on the leech were separated and clear, while it had been reduced to unclear shadows in the previous report. The phylogenetic tree based on the *COI* gene showed that the *COI* gene sequence obtained in this study was in the same evolutionary branch as *Hemiclepsis khankiana* (MN295420, MN295421). Genetically, it was most closely related to *Hemiclepsis kasmiana* (mean COI *p*-distance = 3.98%).

**Conclusions:**

The current study reported on the new distribution range of *Hemiclepsis khankiana*, which was initially discovered in China. This study indicates that the distribution range of the leech species has expanded, laying a foundation for further studies in China.

## Background

Leeches are an integral component of aquatic biocenosis and can be found in a wide range of ecosystems such as freshwater, saltwater, flowing, and still-water ecosystems [1, 2]. It has the largest distribution in coastal areas, especially plays an important role in the freshwater benthic community in coastal areas, and is an important part of the food web [3]. In addition to the above-mentioned roles, leeches are accumulators of toxic substances and biological indicators of water pollution and are also directly related to the spread of bacterial and viral infections [2].

The freshwater leech genus *Hemiclepsis* Vejdovsky, 1884 (Hirudinea: Glossiphoniidae) currently contains at least 16 species, five of which have been recently discovered. Among them, four species were found in Russia in 2019, namely *Hemiclepsis khankiana*, *Hemiclepsis myanmariana*, *Hemiclepsis schrencki* and *Hemiclepsis tumniniana*, and one species *Hemiclepsis yangtzenensis* was found in China in 2021 [4, 5]. Bolotov et al. [4] found that *Hemiclepsis* originated from East Asia, and the Asian *Hemiclepsis* mussel-associated leeches have had a wide range of ancestors in both East Asia and Southeast Asia. Most of the reported species belonging to the genus *Hemiclepsis* are mainly distributed in some countries on the Asian continent, such as China, India, Japan, South Korea and Russia, and the hosts are mainly freshwater leeches and freshwater fish [4, 6]. In China, six species of *Hemiclepsis* have been reported: *H. erhaiensis*, *H. guangdongensis*, *H. hubeiensis*, *H. kasmiana, H. marginata* and *H. yangtzenensis*. Among this species, *H. erhaiensis* and *H. hubeiensis* were found on the gills of freshwater fish, *H. guangdongensis* was collected from the skin of *Cuora amboinensis*, *H. kasmiana* and *H. yangtzenensis* were collected from the body surface of *Monopterus albus*, and *H. marginata* also has been found on the body surface of *M. albus* [5–9].

According to Bolotov’s previous report, there were seven stages in the life cycle of *Hemiclepsis* mussel-associated leeches [4]. Firstly, the mature leech leaves the mantle cavity and fixes the egg cluster on the back edge of the host shell near the umbo, and covers the brood with its body to develop the eggs. Then, the eggs hatch into larvae and attach to the ventral surface of the parent, entering the mantle cavity of the host mussel with the parent. Subsequently, the larvae leave the parent body and probably start to feed on the host mussel. The leech larvae grow into adults. Proposed feeding on the host mussel (still to be confirmed). Finally, adult leeches grow into maturation leeches feeding on freshwater fish as their primary host. From the above description of life history, it can be found that most stages in the life cycle of *Hemiclepsis* mussel-associated leeches mainly occur in the mantle cavity of the mussel.

*Hemiclepsis khankiana* is a new species that belonging to the genus *Hemiclepsis,* discovered in the Russian Far East in 2019 [4]. This species was considered a possible obligate inhabitant of the mantle cavity of freshwater mussels [4]. Bolotov et al. [4] discovered that the secondary host and shelter of this species were freshwater mussels *Nodularia* (Unionidae: Unioninae), and the primary host were freshwater fishes *Rhodeus* (Cyprinidae), the distribution ranges were in the Khanka Lake Basin and Amur Basin system, Russia Far East and probably China. However, there have been no recent reports of this species in China [4].

Freshwater mussels have become one of the most important fauna in freshwater ecosystems due to their potential to enhance biodiversity and ecosystem functions [10–14]. China is one of the countries with the highest species diversity of freshwater mussels, especially the Yangtze River is the center of freshwater mussel diversity in East Asia [15]. As a province rich in water resources in the upper reaches of the Yangtze River, Sichuan has developed freshwater aquaculture industries such as fish, crustaceans and shellfish. Recently, a freshwater leech species was found in the mantle cavity of freshwater mussels in Zigong, Sichuan Province. In the current study, this experiment observed the morphology of the freshwater leech by stereomicroscope and scanning electron microscope and identified the characterization by molecular biology methods. Finally, the distribution range, morphological differences, and phylogenetic analysis of this species were discussed.

## Results

### Morphological analysis

**Fig. 1:**
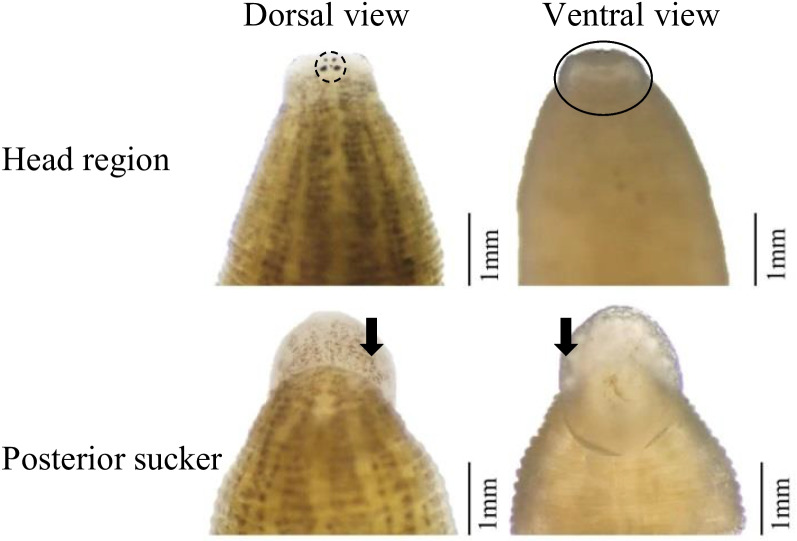
External morphology of *Hemiclepsis khankiana*’s head region (dorsal view and ventral view) and posterior sucker (dorsal view and ventral view). The part circled by the dotted line represents the two pairs of eyespots of the leech. The part circled by the solid line represents the oral sucker (ventral view) of the leech. Black arrows indicate the posterior sucker (dorsal view and ventral view) of leech

**Fig. 2:**
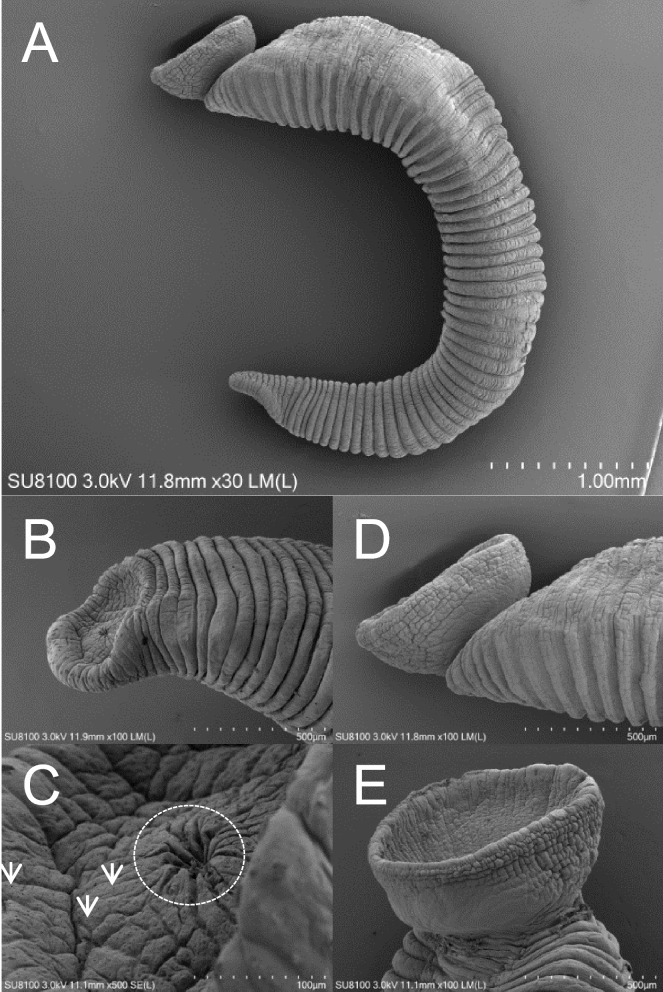
Scanning electron photomicrographs of *Hemiclepsis khankiana*. (**A**) Side view of the *Hemiclepsis khankiana*. (**B**) and (**C**) Side view of the oral sucker. (**D**) and (**E**) Side view of the caudal sucker. The part circled by the dotted line represents the stellate, radial pit of the leech oral sucker. The white arrows indicate small pore structures of the leech oral sucker

In this study, all of the 48 freshwater mussel (Unionidae: Unioninae) specimens were examined, with 19 freshwater mussels being infested by 84 leeches. The leech observed in this study was a medium-sized rice-shaped leech with dorsoventrally flatted and dorsum smoothly. The body length of the representative specimen from the anterior tip to the rear posterior sucker was 7.85 mm, the body widest point was 2.21 mm, and the posterior sucker diameter was 1.69 mm, broader than the oral sucker width (1.12 mm). The characteristics of leech samples could be summarized as follows: the dorsal of the leech samples was pale yellow, with six vertical, widely brown stripes; the posterior sucker had no strips but with dense and diffuse brown spots. Two pairs of obvious eyespots, the first pair of eyespots was small but visible and was located ahead of the second pair of eyes, both pairs were drop-like and separated (Fig. 1). Scanning electron microscopy provided *H. khankiana* surface morphology structures (Fig. 2). The photographic evidence showed a small oral sucker (Fig. 2B and C) and a large deeply cupped posterior sucker ((Fig. 2D and E)). In the center of the oral sucker was a stellate, radial pit (Fig. 2C). The surface of the whole oral sucker was full of small pore structures (Fig. 2C).

### Molecular and phylogenetic analyses

**Fig. 3:**
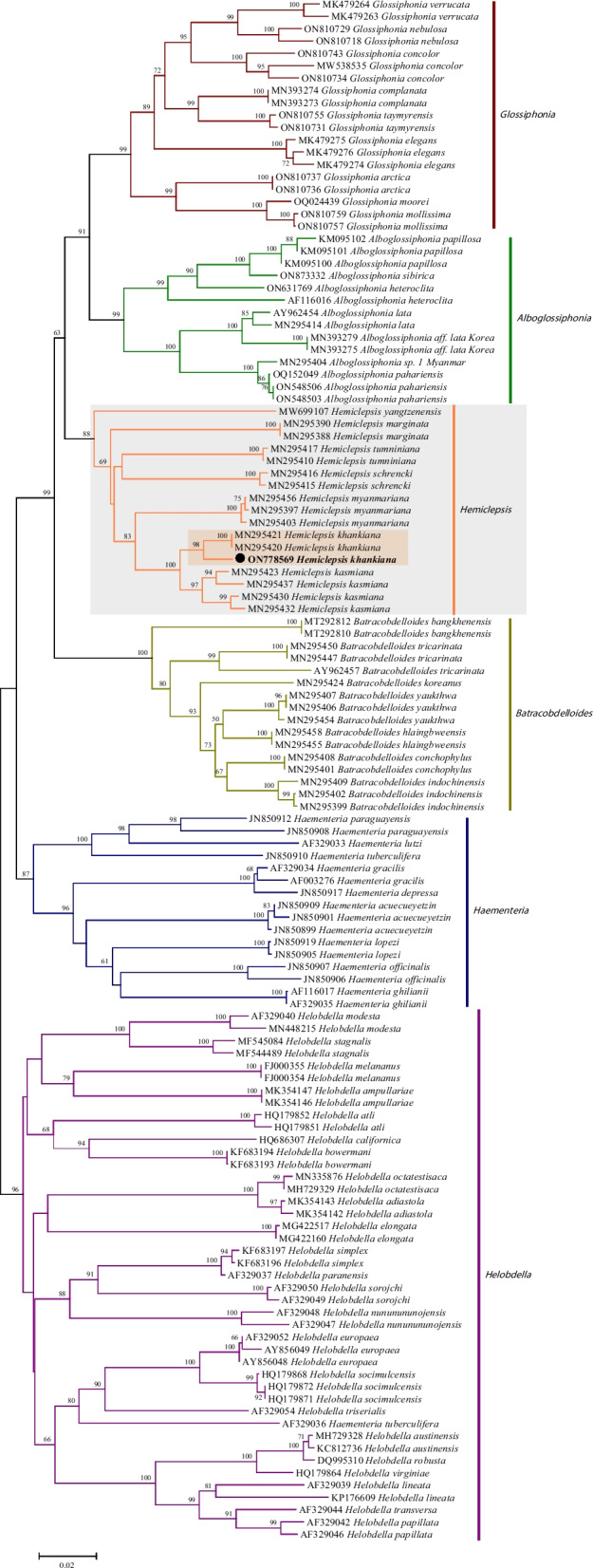
Phylogenetic tree of the leech based on the *COI* gene sequences. The tree was constructed with the neighbor-joining method using MEGA 7.0. Numbers at nodes represent the percentage occurrence of clades in 1000 bootstrap replications of data. The solid circle indicates the sequence from this study. All sequence from this study was bolded

**Fig. 4:**
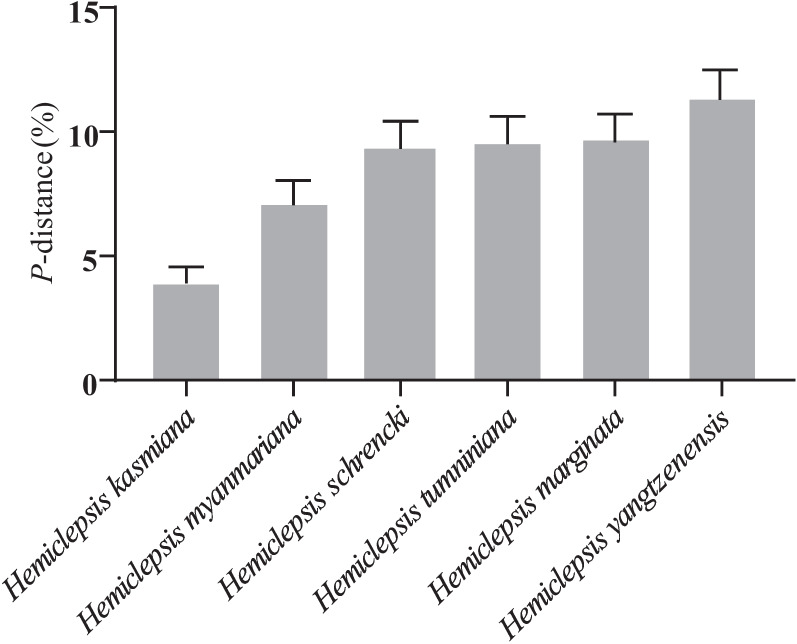
Genetic distances between *Hemiclepsis khankiana* and other same genus species based on the *COI* gene. The bars indicate standard error estimates of the distance values based on the bootstrap approach (1000 replications)

The BLASTn analysis showed that the *COI* partial sequence (ON778569) obtained in the current study had 98.03% identity with the *H. khankiana COI* sequence (MN295420) in Russia. Phylogenetic analysis revealed that *COI* partial sequence (ON778569) was in the same clade as *H. khankiana* (MN295420 and MN295421) isolated from Russia (Fig. 3). Genetic distance among various *Hemiclepsis* clades was 3.98–12.83% and *H. khankiana* with 3.98–11.41% genetic distance with other *Hemiclepsis* species. Genetically, this *Hemiclepsis* species was most closely related to *H. kasmiana* (mean *COI p*-distance = 3.98%) (Fig. 4).

## Discussion

The current study described a freshwater leech species parasitic in the freshwater mussel mantle cavity. This species was identified as *H. khankiana* by morphological analysis and molecular biology. This *Hemiclepsis* species also has been reported for the first time in China.

**Fig. 5:**
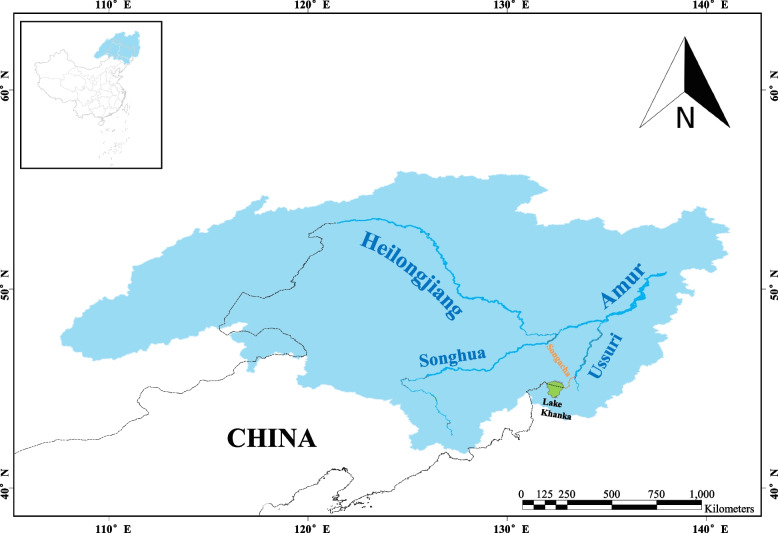
The geographical location of the Heilongjiang (Amur) River Basin (the blue filling)

*Hemiclepsis khankiana*, a new *Hemiclepsis* species that was discovered in recent years, had only one record. The name of this species was derived from the Khanka Lake basin in the Russian Far East [4]. As shown in Fig. 5, Khanka Lake is the boundary lake between China and Russia, which lies on the border of Primorsky Krai (Russia) and the Heilongjiang province of China. Its only outflow is the Songacha (Song’acha) River, a tributary of the Ussuri (Wusuli) River. Then, the Ussuri (Wusuli) River heads north to join the Amur River near Khabarovsk. Lake Khanka belongs to the Ussuri River System, which is part of the Amur River System. The Amur Basin is also called the Heilongjiang River Basin in China. This suggested that *H. khankiana* may also exist in the Heilongjiang River Basin, China. In addition, the primary host of *H. khankiana* is the freshwater fish *Rhodeus* (Cyprinidae), and *Rhodeus* is widely distributed in China, as well as in the Yangtze River Basin and Heilongjiang River Basin [16]. Although in this study, this species was collected in the Yangtze River Basin and has not been reported in the Heilongjiang River Basin, this investigation confirmed Bolotov’s guess that *H. khankiana* may also be distributed in China, and updated and expanded the distribution range of *H. khankiana*. At present, it is unclear whether the distribution of this species in China was caused by natural factors or factors such as the transportation of aquatic products, and whether the expansion of the species would have an impact on freshwater mussels and freshwater fish in the distribution area.

The dorsal view of the leech sample head region (Fig. 2), showed that the sample collected in this study was different in the description of the eyespots of *H. khankiana*. Bolotov et al. [4] described eyespots of *H. khankiana* as “the first pair strongly reduced up to an unclear shading, the second pair was cup-like”, but from observed in this study, the first pair of eyes was very obvious, and the second pair of eyes closer to the shape of water drops (Fig. 2). The same species had stable morphological characteristics, This result suggested the phenotype of a species was the result of the interaction between its genotype and the environment. Whether the difference between the morphological characteristics of *H. khankiana* described in this study and the Bolotov et al. [4] reported was caused by environmental factors was unknown, and further information mining may be required for this species.

The phylogenetic tree and genetic distance analysis of the *COI* gene showed the evolutionary relationship clearly between *H. khankiana* and other *Hemiclepsis* species. Among them, *H. khankiana* and *H. kasmiana* were closely related in evolution, and they were in a large evolutionary branch, with a bootstrap value of up to 98. Moreover, the mean COI *p*-distance between *H. khankiana* and *H. kasmiana* was the closest, with a value of 3.8%. In addition, we found that *H. yangtzenensis*, a new species of *Hemiclepsis* species discovered in 2021, was significantly different from other *Hemiclepsis* species, and had a relatively far genetic distance (mean *COI p-*distance = 10.41–12.83%) from other species in the analysis of genetic distance [5].

Previous studies have shown that global mussel-associated leech assemblage includes at least 12 species belonging to the family Glossiphoniidae [4]. Mussel-associated leech taxa have been reported in East and Southeast Asia, India, Nepal, and Africa, but have not been reported in Europe, the Middle East, North and Central Asia, Australia, the Indonesian archipelago, New Guinea, and the Philippines [4]. Research on the genera *Batracobdelloides* and *Hemiclepsis* were largely overlooked by researchers, and the number of *Hemiclepsis* species in China was still very small, but with the discovery of the new species, we are still largely underestimating the number of *Hemiclepsis* species [5]. The freshwater leech genus *Hemiclepsis* has great value in respect of aquaculture matters. *M. albus* infection with a large amount of *H. marginata* will cause massive blood loss of *M. albus*, leading to *M. albus* dying in large numbers [17]. In addition, *H. marginata* is also an important vector for the spread of fish diseases, such as trypanosomes and pancreatic necrosis viruses [18–20]. *H. khankiana* also is parasitic on freshwater fish, but whether the species can have the ability to spread the pathogenic to the host like other freshwater leeches needs further exploration. Whether a large number of *H. khankiana* parasites can be harmful to fish has not been reported. In short, there is currently only one relevant report about *H. khankiana*, and more information about it needs further research.

So far, only a few species have been described in the world, and the actual number of invasive species may exceed the current record and will further increase. For newly discovered species or alien species that have not been understood by science, their functions have not been fully observed or understood [21, 22]. There are six broad mechanisms for alien species to be introduced into a region, among which ‘contaminant’ and ‘stowaway’ are common for invertebrates, algae, fungi and microorganisms, because these organisms are widespread and inconspicuous, and are often introduced through transportation. For example, pathogens and parasites are frequently introduced as contingents with their hosts [23–26]. In China, the invasion of *Pomacea canaliculata* has not only caused serious economic losses to local agricultural production, wetland ecosystems and freshwater resources but also served as an intermediate host for many human and livestock parasites and pathogens, the most famous of which is *Angiostrongylus cantonensis*, which can cause diseases such as eosinophilic meningitis or meningoencephalitis when infecting humans [27–29].

The impact of exotic parasites and pathogens is usually hidden [30]. Even if a species was detected in its alien range, even though its ecological function and changes are not mysterious, it may still not be found if it is affected by seasonal, annual or regional variations, temporal and spatial variations and human beings. Pathogens can also evolve rapidly, so many exotic pathogens with serious impacts are not considered threats before they are established in new hosts [30]. Moreover, the transmission of parasites needs to reach a certain population density and the specific space-time conditions required for transmission [31, 32]. The freshwater leech parasitic on freshwater mussels found in this study was reported for the first time in China. It cannot be ruled out that the species invaded China through natural factors or freshwater aquatic product trades between Russia and China, and other factors.

With the rapid growth of world maritime trade, the world is connected by shipping corridors. Two of these, the Panama and Suez canals, are major shipping corridors that are considered hotspots for biological invasions [33]. Some species of halobios can freely shuttle between the two ends of the canal and become exotic organisms in another sea area. In recent years, a variety of fish has entered the Mediterranean through the Suez Canal, increasing the number of species in the Mediterranean by 8%. In addition, a variety of previously unreported marine fish has also been found in Gatun Lake in Panama. The number of some species in Gatun Lake has increased significantly, almost completely replacing the previously existing freshwater fish in the lake [34]. Although the primary host of freshwater leech parasitized on freshwater mussels found in this study is widely distributed in China, this does not exclude that this species may invade China through natural factors such as “corridor”. After all, the Amur River basin and the Heilongjiang River basin belong to the same water system. Of course, this problem needs further study.

## Conclusions

In conclusion, firstly, the current study reported the new distribution range of *H. khankiana*, this species was found in China. This study also gave a simple description of *H. khankiana* in morphology. It was necessary to further survey the host range of *H. khankiana* and whether H. *khankiana* transmits pathogens and can harm its host.

## Methods

### Sample collection

**Fig. 6:**
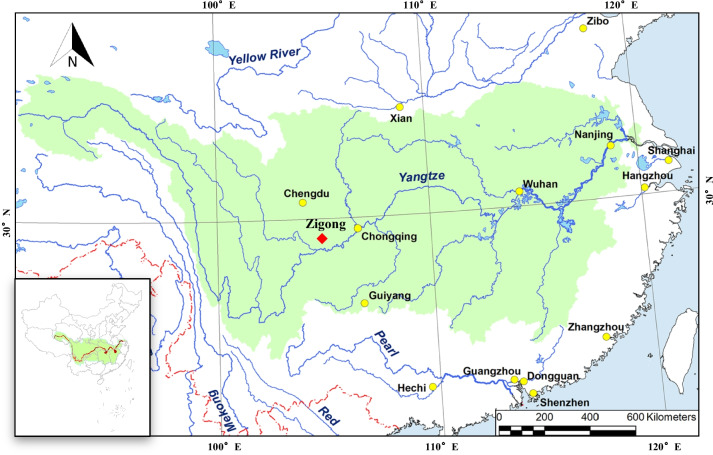
The geographical location of the sample sampling (red solid diamond). The green filling indicates the Yangtze River basin. (The figure was modified based on the map in the article by XU et al., 2021 [5])

The leech specimens were collected in the mantle cavity of freshwater mussels of the Zigong (29°21′49.36″N, 104°46′26.04″E), Sichuan Province, China (Fig. 6). The collected samples were partially stored at − 80 °C for the following experiments, and partly stored in absolute ethanol for observation under the microscope. The remaining samples were stored in the electron microscopy fixative for scanning electron microscope.

### Morphological analyses

In this study, the leech samples were observed by the stereoscopic microscope. According to the key of mussel-associated leech species (Glossiphoniidae: *Batracobdelloides* and *Hemiclepsis*) written by Bolotov et al. [4], we mainly observed the color and pattern of the dorsum of the leeches, the size and color of the suckers, the shape and number of the eyespots, and preliminarily determined the species of leeches samples according to the external morphological characteristics through the above observation results [4].

To better observe the external characteristics of the leech samples, the current study also observed them by scanning electron microscopy, which was briefly described as follows: the samples fixed with electron microscopy fixative were washed three times with 0.1 M phosphate buffer (pH 7.4) for 15 minutes each. Then, the samples were transferred to a 1% OsO_4_ solution prepared with a 0.1 M phosphate buffer (pH 7.4) and incubated at room temperature for 1-2 hours. After that, the samples were washed in 0.1 M phosphate buffer (pH 7.4) three times for 15 minutes each. Then, the samples were sequentially placed in 30%, 50%, 70%, 80%, 90%, 95%, 100% and 100% alcohol for dehydration, with each step lasting 15 minutes. Then, isoamyl acetate was used to exchange the ethanol for 15 minutes. Dried samples with a Critical Point Dryer, then sputter-coated with gold for 30 seconds, and finally digital images were captured using a scanning electron microscope.

### Molecular analyses

The mitochondrial cytochrome c oxidase subunit I gene (*COI*) sequence, known as an available DNA barcoding marker for animals, is frequently used for phylogenetic estimation of leeches [35, 36]. Therefore, we selected *COI* as the target gene to be amplified by polymerase chain reaction (PCR). The *COI* partial sequence for the leeches was obtained using the following primers: LoboF1 (5′-KBT CHA CAA AYC AYA ARG AYA THG G-3′) and LoboR1 (5′-TAA ACY TCW GGR TGW CCR AAR AAY CA-3′) [37]. Total genomic DNA was extracted from samples that were stored -80 °C using the TIANamp Genomic DNA Kit (TIANGEN, China), following the manufacturer’s protocol. PCR reaction volume of 25 μL containing 3 μL of DNA template, 0.5 μL each of forward and reverse primers (10 μM), 0.25 μL of *Taq* polymerase (0.5 U; New England BioLab, USA), 1.25 μL of deoxyribonucleotide triphosphate (200 μM; New England BioLab, USA), 2.5 μL of 10 × ThermoPol Reaction Buffer (New England BioLab, USA), and double-distilled water to a total volume of 25 μL. PCR reaction programs for *COI* are as follows: 95 °C (5 min), followed by 35 cycles at 95 °C (50 s), 48 °C (50 s), 68 °C (1 min), and a final extension at 68 °C (5 min). PCR product was purified using EasyPure® Quick Gel Extraction Kit (TransGen, China) and cloned into *E. coli* DH5α using the PMD™ 19-T Vector Cloning Kit (TaKaRa, Japan). At least two positive clones were selected for sequencing at Sangon Biotech (Shanghai) Co., Ltd.

### Sequence and phylogenetic analyses

Nucleotide sequence identities were determined by performing GenBank BLASTn analysis (https://blast.ncbi.nlm.nih.gov/Blast.cgi). The sequence was then submitted to GenBank to obtain the accession number. For phylogenetic analyses, the newly obtained sequence of *H. khankiana* (ON778569) was added. Further more, we obtained several published *Hemiclepsis* COI sequences as follows: *Hemiclepsis kasmiana* (MN295423, MN295430, MN295432, MN295437); *H. khankiana* (MN295420, MN295421); *H. myanmariana* (MN295403, MN295456, MN295397); *Hemiclepsis marginata* (MN295390, MN295388); *H. tumniniana* (MN295410, MN295417); *H. schrencki* (MN295415, MN295416); *H. yangtzenensis* (MW699107), and other Glossiphoniidae leeches species.

Based on the above sequences, the Neighbor-Joining method was used to construct a phylogenetic tree, and calculated genetic distance between *H. khankiana* and other *Hemiclepsis* species by using the *p*-distance model in MEGA7.0 [38].

## Data Availability

The datasets generated and analyzed during the current study are available in the NCBI repository, https://www.ncbi.nlm.nih.gov/nuccore/ON778569.

## References

[1] Siciński J, Błaszak C (2009). Annelids Annelida. Zoology: invertebrates.

[2] Fedorova LI, Kaygorodova IA (2022). First data on the hirudinea fauna of lotic ecosystems of the Khanty-Mansi autonomous area (Russia). ZooKeys..

[3] Adamiak-Brud Ż, Bielecki A, Kobak J, Jabłońska-Barna I (2016). Rate of short-term colonization and distribution of leeches (Clitellata: Hirudinida) on artificial substrates. J Zool.

[4] Bolotov IN, Klass AL, Kondakov AV, Vikhrev IV, Bespalaya YV, Gofarov MY (2019). Freshwater mussels house a diverse mussel-associated leech assemblage. Sci Rep.

[5] Xu Z, Yang C, Gofarov MY, Eliseeva TA, Kondakov AV, Yuan H (2021). A new freshwater leech species from Asian swamp eel stocks in China. Parasitol Res.

[6] Bolotov IN, Vikhrev IV, Aksenova OV, Bespalaya YV, Gofarov MY, Kondakov AV (2017). Discovery and natural history of the mussel leech Batracobdella Kasmiana (Oka, 1910) (Hirudinida: Glossiphoniidae) in Russia. Zootaxa.

[7] Yang T (1981). Two new species of parasitic leeches from freshwater fishes in China. Acta Zootaxon Sin.

[8] Yang T (1996). Annelida Hirudinea (Fauna Sinica). Fauna Sinica.

[9] Tan G, Liu Q (2001). One new species of the genus *Hemiclepsis* (Rhynchobdellida: Glossiphoniidae). Acta Zootaxon Sin.

[10] Jones JW, Hallerman EM, Neves RJ (2006). Genetic management guidelines for captive propagation of freshwater mussels (Unionoidea). J Shellfish Res.

[11] Graf DL, Cummings KS (2007). Review of the systematics and global diversity of freshwater mussel species (Bivalvia: Unionoida). J Molluscan Stud.

[12] He J, Zhuang Z (2013). The freshwater bivalves of China.

[13] Liu X, Cao Y, Xue T, Wu R, Zhou Y, Zhou C (2017). Genetic structure and diversity of *Nodularia douglasiae* (Bivalvia: Unionida) from the middle and lower Yangtze River drainage. PLoS One.

[14] Vaughn CC (2018). Ecosystem services provided by freshwater mussels. Hydrobiologia.

[15] Zieritz A, Bogan AE, Froufe E, Klishko O, Kondo T, Kovitvadhi U (2018). Diversity, biogeography and conservation of freshwater mussels (Bivalvia: Unionida) in east and Southeast Asia. Hydrobiologia.

[16] Li F, Arai R, Liao TY (2020). *Rhodeus flaviventris*, a new bitterling (Teleostei: Cyprinidae: Acheilognathinae) from China. Zootaxa.

[17] Liu X, Guo L (2002). The first report of *Monopterus Albus* leech (*Hemiclepsis marginata*) disease. Acta Hydrobiol Sin.

[18] Lewis JW, Ball SJ (1980). Ultrastructure of the epimastigotes of the fish trypanosome *Trypanosoma cobitis* Mitrophanow 1883, in the crop of the leech vector, *Hemiclepsis Marginata*. J Parasitol.

[19] Jones SR, Woo PT (1992). Vector specificity of *Trypanosoma Catostomi* and its infectivity to freshwater fishes. J Parasitol.

[20] Salimi B, Abdi K (2016). Detection of infectious pancreatic necrosis virus from the leeches *Hemiclepsis Marginata* and *Hirudo Medicinalis*. J Aquat Anim Health.

[21] Hendrix PF (2006). Biological invasions belowground—earthworms as invasive species. Biol Invasions.

[22] Ricciardi A, Thorp JH, Rogers DC (2015). Ecology of invasive alien invertebrates. Ecology and general biology: Thorp and Covich’s freshwater invertebrates.

[23] Hulme PE, Bacher S, Kenis M, Klotz S, Kühn I, Minchin D (2008). Grasping at the routes of biological invasions: a framework for integrating pathways into policy. J Appl Ecol.

[24] Perkins SE, Altizer S, Bjornstad O, Burdon JJ, Clay K, Gómez-Aparicio L, Eviner VT, Keesing F, Ostfeld RS (2008). Invasion biology and parasitic infections. Infectious disease ecology: effects of ecosystems on disease and of disease on ecosystems.

[25] Saul W-C, Roy HE, Booy O, Carnevali L, Chen HJ, Genovesi P (2017). Assessing patterns in introduction pathways of alien species by linking major invasion databases. J Appl Ecol.

[26] Pyšek P, Hulme PE, Simberloff D, Bacher S, Blackburn TM, Carlton JT (2020). Scientists' warning on invasive alien species. Biol Rev Camb Philos Soc.

[27] Yii CY (1976). Clinical observations on eosinophilic meningitis and meningoencephalitis caused by *Angiostrongylus cantonensis* on Taiwan. Am J Trop Med Hyg.

[28] Wang QP, Wu ZD, Wei J, Owen RL, Lun ZR (2012). Human Angiostrongylus cantonensis: an update. Eur J Clin Microbiol Infect Dis.

[29] Wang LJ, Xu Y, Sun H, Zhang BG, Kong XL, Han HT (2022). First report of invasive *Pomacea snails* in Shandong Province. Zhongguo Xue Xi Chong Bing Fang Zhi Za Zhi.

[30] Roy HE, Hesketh H, Purse BV, Eilenberg J, Santini A, Scalera R (2017). Alien pathogens on the horizon: opportunities for predicting their threat to wildlife. Conserv Lett.

[31] Torchin ME, Lafferty KD, Dobson AP, McKenzie VJ, Kuris AM (2003). Introduced species and their missing parasites. Nature.

[32] Prenter J, Macneil C, Dick JT, Dunn AM (2004). Roles of parasites in animal invasions. Trends Ecol Evol.

[33] Seebens H, Schwartz N, Schupp PJ, Blasius B (2016). Predicting the spread of marine species introduced by global shipping. Proc Natl Acad Sci U S A.

[34] Castellanos-Galindo GA, Robertson DR, Sharpe DMT, Torchin ME (2020). A new wave of marine fish invasions through the Panama and Suez canals. Nat Ecol Evol.

[35] Siddall ME, Burreson EM (1998). Phylogeny of leeches (Hirudinea) based on mitochondrial cytochrome c oxidase subunit I. Mol Phylogenet Evol.

[36] Tessler M, de Carle D, Voiklis ML, Gresham OA, Neumann JS, Cios S (2018). Worms that suck: phylogenetic analysis of Hirudinea solidifies the position of Acanthobdellida and necessitates the dissolution of Rhynchobdellida. Mol Phylogenet Evol.

[37] Lobo J, Costa PM, Teixeira MA, Ferreira MS, Costa MH, Costa FO (2013). Enhanced primers for amplification of DNA barcodes from a broad range of marine metazoans. BMC Ecol.

[38] Kumar S, Stecher G, Tamura K (2016). MEGA7: molecular evolutionary genetics analysis version 7.0 for bigger datasets. Mol Biol Evol.

